# Clinical spectrum and prognostic features of patients seropositive for anti-GD1a antibody

**DOI:** 10.3389/fimmu.2026.1825556

**Published:** 2026-07-14

**Authors:** Xujun Chu, Xinyu Liu, Ziling Zeng, Jinping Li, Xiaoyu Liu, Shougang Guo, Rui Wu

**Affiliations:** 1 Department of Neurology, Shandong Provincial Hospital Affiliated to Shandong First Medical University, Jinan, Shandong, China; 2 Department of Clinical Psychology, Shandong Provincial Hospital Affiliated to Shandong First Medical University, Jinan, Shandong, China; 3 Department of Laboratory, The People’s Hospital of Huaiyin, Jinan, Shandong, China

**Keywords:** AMAN, AMSAN, anti-GD1a antibody, CIDP, immunotherapy, MFS

## Abstract

**Background:**

Anti-GD1a antibodies are associated with a range of immune-mediated neuropathies, particularly acute motor axonal neuropathy (AMAN) and other Guillain-Barré syndrome (GBS) variants. However, their full clinical spectrum and prognostic significance remain unclear due to limited systematic studies. Here, we investigated the clinical phenotypes, serological profiles, cerebrospinal fluid (CSF) characteristic, and therapeutic outcomes associated with anti-GD1a antibodies.

**Methods:**

The clinical, paraclinical and therapeutic data were retrospectively collected and analyzed from 19 Chinese patients who tested positive for anti-GD1a antibodies.

**Results:**

The mean age at onset in this cohort was 50.6 years. Among the 19 patients, 14 (74%) presented with acute syndromes. The remaining five patients (26%) exhibited features consistent with chronic neuropathies. Common manifestations included absent tendon reflexes (84%), limb weakness (84%), sensory impairment (58%), and cranial nerve involvement (53%). Isolated anti-GD1a antibody positivity was observed in only 5 patients, while others had co-existing antiganglioside antibodies, most commonly anti-GQ1b, and anti-GM1. Electrophysiological studies revealed axonal and demyelinating neuropathic patterns. Patients with cranial nerve involvement and albuminocytologic dissociation were associated with higher modified Erasmus GBS Outcome Score (mEGOS). Immunotherapy was effective in acute cases, with 57% achieving complete recovery at 6-month follow-up.

**Conclusions:**

Patients seropositive for anti-GD1a antibody exhibits significant clinical and electrophysiological heterogeneity and frequently coexists with other antiganglioside antibodies. Acute presentations generally respond well to immunotherapy, while chronic cases require individualized management.

## Introduction

Guillain-Barré syndrome (GBS) and its related disorders are a heterogeneous group of acute immune-mediated neuropathies, often preceded by infections and characterized by rapidly progressive sensory, motor and autonomic dysfunction (1). The pathogenesis of these conditions is strongly associated with antibodies directed against gangliosides, which are glycosphingolipids abundantly expressed in the peripheral nervous system (2). Among these, antibodies such as GM1, GQ1b and GT1a have been well characterized and correlate with specific clinical phenotypes (2). Several antibody-associated subtypes of GBS have been defined by their clinical features, including ‘typical’ forms such as motor-sensory and pure motor GBS, as well as GBS variants and Miller Fisher syndrome (MFS) spectrum (3).

Among the various antiganglioside antibodies, anti-GD1a antibodies have been particularly implicated in the pathogenesis of peripheral neuropathies, especially in acute motor axonal neuropathy (AMAN) (4, 5), multiple cranial nerve palsy (6) and ophthalmoplegia (7). This clinical association may relate to the specific localization and function of GD1a. The GD1a is expressed at the motor nerve terminal and axonal membrane, making it a plausible target for immune-mediated attack leading to axonal dysfunction (8). Besides, GD1a is also one of the major ligands for myelin-associated glycoprotein (9). Existing knowledge about anti-GD1a antibodies is based on case reports or small series, which describe associations with acute motor-sensory deficits and cranial nerve involvement (6–8). In addition to their diagnostic value, serum anti-GD1a IgG antibodies have also been suggested as potential prognostic markers, especially for predicting poor outcomes (10). However, the full clinical and electrophysiological spectrum of anti-GD1a antibodies-associated syndromes remains incompletely defined. Moreover, anti-GD1a antibodies have occasionally been reported in chronic neurological conditions (1, 11), though their pathological significance in these contexts is still unclear.

Given the limited systematic data on the clinical and prognostic features of anti-GD1a antibodies syndromes, we conducted a retrospective study of 19 patients with confirmedanti-GD1a antibody positivity. This study aimed to comprehensively characterize the clinical features, paraclinical findings including serological, electrophysiological and cerebrospinal fluid (CSF) analyses and treatment responses in this cohort. Our findings aim to improve the recognition of patients seropositive for anti-GD1a antibody and contribute to a deeper understanding of its diagnostic and therapeutic implications.

## Patients and methods

### Patients

A total of 19 patients (10 females, 9 males) with anti-GD1a antibodies were enrolled between January 2022 and June 2025 (**Table 1**). Written informed consent was obtained from all patients. All patients were recruited from individuals presenting to the Department of Neurology of our hospital with acute or progressive limb weakness, and with a clinical suspicion of GBS or other immune-mediated neuromuscular diseases. Patients were consecutively recruited from the Emergency Department (initial presentation and admission) and the inpatient Neurology ward. Inclusion criteria: (1) Age ≥18 years; (2) Acute or progressive limb weakness; (3) Positive serum anti-GD1a antibody (IgG or IgM). Exclusion criteria: (1) Definite diagnosis of other neuromuscular diseases (e.g., myasthenia gravis, polymyositis); (2) Evidence of central nervous system lesions (e.g., stroke, myelitis); (3) Clinical or laboratory evidence suggestive of porphyria, lead poisoning, or botulism. Clinical data were retrospectively analyzed regarding age, sex, duration of disease, history of diarrhea or symptoms of an upper respiratory tract infection within 4 weeks preceding onset of weakness, cranial nerve damage, degree of muscle weakness, sensory disturbance, absent deep tendon reflexes, autonomic dysfunction, cerebellar ataxia, and pathological reflexes. According to whether the time from onset to peak exceeded 21 days, patients seropositive for anti-GD1a antibody were classified as acute and chronic course. Patients seropositive for anti-GD1a antibody with acute course were further classified into six subtypes: (1) AMAN, (2) acute motor sensory axonal neuropathy (AMSAN), (3) acute inflammatory demyelinating polyneuropathy (AIDP), (4) MFS, (5) Bickerstaff brainstem encephalitis (BBE), and (6) pharyngeal-cervical-brachial (PCB) variant. For these patients with acute course, the Medical Research Counsel (MRC) sumscore, GBS disability score, and the modified Erasmus GBS Outcome Score (mEGOS) were calculated to assess disease severity. The MRC sumscore is calculated as the sum of MRC scores of 6 different muscles (abduction of the arm, flexion of the forearm, extension of the wrist, flexion of the leg, extension of the knee, dorsal flexion of the foot) measured bilaterally, which results in a sumscore ranging from 0 (paralysis) to 60 (normal strength (12). The GBS disability score is used for assessing the functional status of patients on a scale from 0 (healthy) to 6 (dead) (13). The mEGOS is calculated based on age (0 to 3), presence of diarrhea (0 to 1) and MRC sum score (0 to 6). Higher mEGOS predicted poorer outcomes (14).

**Table 1:** Clinical features of patients seropositive for anti-GD1a antibody.

No.	Sex	Diagnosis	Age	Course	Preceding infection	Cranial nerve damage	Ophthalmoparalysis	Muscle weakness	Tendon reflex	Sensory disturbance	Superficial sense impairment	Deep sense impairment	Pain	Cerebellar ataxia	Pathologic reflex	Autonomic dysfunction	Mechanical ventilation
1	Male	CIDP	68	7 years	No	No	No	Yes	Absent	No	No	No	No	No	No	No	No
2	Male	AMSAN	51	7 days	Yes	Yes	Yes	Yes	Absent	Yes	No	No	No	Yes	No	No	No
3	Male	AMAN	32	7 days	Yes	No	No	Yes	Dcreased	Yes	No	No	Yes	No	No	No	No
4	Male	AMAN	63	5 days	Yes	No	No	Yes	Dcreased	Yes	No	No	Yes	Yes	No	No	No
5	Female	POEMS	31	300 days	Yes	No	No	Yes	Dcreased	Yes	Yes	Yes	Yes	No	No	Yes	No
6	Female	AMAN	57	12 days	Yes	Yes	No	Yes	Dcreased	No	No	No	No	No	No	No	No
7	Female	AIDP	75	8 days	Yes	Yes	Yes	Yes	Dcreased	Yes	Yes	No	No	No	No	No	No
8	Female	PN	37	4 years	No	No	No	Yes	Dcreased	No	No	No	No	No	No	No	No
9	Male	sensory neuropathy associated with SS	48	7 years	No	No	No	No	Dcreased	Yes	Yes	Yes	No	No	No	No	No
10	Male	MFS	68	9 days	No	Yes	Yes	No	Dcreased	Yes	No	No	Yes	Yes	No	No	No
11	Female	ALS	55	190 days	No	Yes	No	Yes	Brisk	Yes	Yes	No	No	No	Yes	No	No
12	Female	MFS	55	4 days	Yes	Yes	Yes	Yes	Absent	Yes	Yes	Yes	No	Yes	No	No	No
13	Female	MFS	81	7 days	Yes	No	No	No	Dcreased	Yes	No	No	Yes	Yes	No	No	No
14	Male	AMAN	68	4 days	Yes	Yes	No	Yes	Absent	No	No	No	No	No	No	No	Yes
15	Male	AMAN	58	14 days	Yes	Yes	No	Yes	Dcreased	No	No	No	No	No	No	No	Yes
16	Male	PCB variant	18	7 days	Yes	Yes	No	Yes	Dcreased	No	Yes	No	No	No	No	No	No
17	Male	AMAN	37	6 days	No	No	No	Yes	Dcreased	No	No	No	No	No	No	No	No
18	Female	AIDP	37	15 days	No	No	No	Yes	Normal	No	No	No	No	No	No	Yes	No
19	Female	BBE	22	18 days	No	Yes	Yes	Yes	Brisk	Yes	Yes	No	No	Yes	Yes	No	No
Positive proportion	–	–	–	–	58%	53%	26%	84%	–	58%	37%	16%	26%	32%	11%	11%	11%

AIDP, acute inflammatory demyelinating polyneuropathy; ALS, amyotrophic lateral sclerosis; AMAN, acute motor axonal neuropathy; AMSAN, acute motor sensory axonal neuropathy; BBE, Bickerstaff brainstem encephalitis; CIDP, chronic inflammatory demyelinating polyradiculoneuritis; MFS, Miller Fisher syndrome; PCB, pharyngeal-cervical-brachial; PN, peripheral neuropathy; SS, Sjögren’s syndrome.

### Serological investigations

We retrospectively collected serological data of 19 patients. Serum IgG and IgM antibodies against gangliosides GM1, GM2, GM3, GM4, GD1a, GD1b, GD3, GQ1b, GT1a, GT1b and sulfatide were detected using western blot, as previously described (15). Serum IgG antibodies were tested in 19 patients for GD1a, in 6 for GQ1b and GT1a, in 5for GM1, in 2 each for GD1b, GT1b, and GM2, and in 1 each for GM3, GM4, GD3, and Sulfatide. (**Table 2**).

**Table 2:** investigations of patients seropositive for anti-GD1a antibody.

No.	Diagnosis	Delay between disease onset and CSF analysis	Mean protein content (g/l)	CSF cell count	Mean CSF white cells (/mm3)	Albuminocytologic dissociation	Antibodies	Titers of GD1a antibodies	Delay between disease onset and NCS	NCS	F wave	Treatment
1	CIDP	7 years	0.39	2	2	No	anti-GD1a IgG anti-GM1 IgM	+	7 years	Axonal and Demyelinating	abnormal	methylprednisolone
2	AMSAN	9 days	0.65	2	2	Yes	anti-GD1a IgG anti-GQ1b IgG	++	9 days	Axonal	normal	IVIg
3	AMAN	8 days	0.43	2	2	No	anti-GD1a IgG anti-GD1b IgG anti-GT1a IgG	++	9 days	Axonal	abnormal	IVIg+methylprednisolone
4	AMAN	15 days	0.58	8	3	Yes	anti-GD1a IgG anti-GM1 IgM anti-GT1a IgG	+++	–	–	–	IVIg+methylprednisolone
5	POEMS	2 months	0.83	2	1	Yes	anti-GD1a IgG	+	2 months	Axonal and Demyelinating	abnormal	Bortezomib + dexamethasone
6	AMAN	15 days	0.43	0	0	No	anti-GD1a IgG anti-GD1b IgG anti-GD3 IgG anti-GT1b IgG	++	11 days	Axonal and Demyelinating	abnormal	IVIg+methylprednisolone
7	AIDP	–	–	–	–	–	anti-GD1a IgG anti-GT1a IgG	++	10 days	Demyelinating	abnormal	IVIg
8	PN	–	–	–	–	–	anti-GD1a IgG	+	4 years	Demyelinating	abnormal	Vitmin B12
9	SS associated sensory neuropathy	7 years	0.32	7	7	No	anti-GD1a IgG	+	7 years	Axonal	normal	methylprednisolone
10	MFS	–	–	–	–	–	anti-GD1a IgG anti-GQ1b IgG anti-Sulfatide IgG anti-Sulfatide IgM	+++	7 days	Axonal	normal	IVIg+methylprednisolone
11	ALS	6 months	0.64	2	2	Yes	anti-GD1a IgG anti-GM2 IgM	+	6 months	Axonal	abnormal	Riluzole
12	MFS	10 days	0.62	15	14	No	anti-GD1a IgG anti-GM4 IgG anti-GT1a IgG anti-GQ1b IgG	++	6 days	Axonal	normal	IVIg
13	MFS	–	–	–	–	–	anti-GD1a IgM	++	10 days	Demyelinating	abnormal	IVIg
14	AMAN	17 days	1.14	3	3	Yes	anti-GD1a IgG anti-GM1 IgG anti-GM3 IgM anti-GQ1b IgG	++	8 days	Axonal and Demyelinating	abnormal	IVIg
15	AMAN	14 days	2.36	7	7	Yes	anti-GD1a IgM anti-GD1a IgG anti-GM1 IgM anti-GQ1b IgG	++	–	–	–	IVIg
16	PCB	9 days	0.42	8	1	No	anti-GD1a IgM anti-GM2 IgM anti-GT1a IgG anti-GT1b IgG anti-GQ1b IgG	+	9 days	normal	normal	efgartigimod
17	AMAN	–	–	–	–	–	anti-GD1a IgG anti-GM1 IgG	++	5 days	Axonal	normal	IVIg
18	AIDP	19 days	0.49	2	1	Yes	anti-GD1a IgG anti-GT1a IgG anti-GT1a IgM	+	14 days	Demyelinating	abnormal	methylprednisolone
19	BBE	19 days	0.61	104	102	No	anti-GD1a IgG	++	–	–	–	methylprednisolone

AIDP, acute inflammatory demyelinating polyneuropathy; ALS, amyotrophic lateral sclerosis; AMAN, acute motor axonal neuropathy; AMSAN, acute motor sensory axonal neuropathy; BBE, Bickerstaff brainstem encephalitis; CIDP, chronic inflammatory demyelinating polyradiculoneuritis; IVIg, intravenous immunogloblin; MFS, Miller Fisher syndrome; NCS, nerve conduction studies; PCB, pharyngeal-cervical-brachial; PN, peripheral neuropathy; SS, Sjögren’s syndrome.

“+” means low titer, “++”means medium titer, “+++” means high titer.

### Other paraclinical investigations

Nerve conduction studies (NCS) were performed in 16 patients. The compound muscle action potentials (CMAPs) were recorded from the median, ulnar tibial, and peroneal. Sensory nerve action potentials (SNAPs) were recorded from the median, ulnar and sural nerve. The categories include axonal and demyelinating patterns. The definitions of demyelinating and axonal cite Chinese Society of Neurology’s literature (16) (**Table 3**).Temporal dispersion and conduction block concepts as signs of demyelination are mentioned in Methods section. CSF analysis was performed using standard methods. (**Table 2**).

**Table 3:** Neuro-electrophysiological features of patients seropositive for anti-GD1a antibody.

Nerves	Parameters	1	2	3	5	6	7	8	9	10	11	12	13	14	16	17	18	Normal values
Median motor nerves	DML, ms	**5.2**	3.9	**4.5**	**4.3**	3.1	**8.5**	**5.4**	3.7	3.2	**4.2**	**6.6**	**4.9**	4.0	3.85	**5.7**	**4.8**	<4
	CMAP, mV	**0.6**	6.0	**0.9**	6.2	**0.7**	**2.1**	**0.4**	10.2	10.2	**2.1**	6	6	**2.8**	6.2	**0.3**	5.5	>5
	MCV, m/s	**19.9**	55.8	59.4	**35.7**	51.1	52.7	**10.1**	52.6	52.7	56.7	55.1	**47.3**	56.4	58.9	52.1	**38.4**	>50
Ulnar motor nerves	DML, ms	**4.0**	2.8	**3.3**	**3.3**	**NR**	**3.5**	**6.6**	**3.1**	2.3	3	2.4	**4.6**	**3.5**	2.3	**3.1**	**3.9**	<3
	CMAP, mV	**0.2**	3.5	**1.8**	9.8	**NR**	5.4	**0.1**	10.6	7.5	2.8	5.8	6.6	**1.5**	5.6	**0.7**	7.3	>4
	MCV, m/s	**46.9**	57.9	55.6	**36.2**	**NR**	55.7	**4**	63.2	62.2	55.1	59.4	60.7	**46.9**	52.0	53.9	52.8	>50
Peroneal motor nerves	DML, ms	3.7	4.7	**5.7**	4.6	4.8	5.0	**NR**	5	3.2	3.7	**3.6**	**7.3**	4.0	4.7	**NR**	**15**	<5.3
	CMAP, mV	**0.26**	1.5	**0.3**	**0.9**	**0.5**	4.1	**NR**	5.6	5.8	2.9	**1.7**	**1.9**	3.7	4.8	**NR**	**1.5**	>2
	MCV, m/s	**28.7**	45.6	42.1	**30.6**	**31.2**	**33.2**	**NR**	42.6	62.2	50.4	45.3	**33.1**	45.4	51.1	**NR**	**35.9**	>40
Tibial motor nerves	DML, ms	3.7	3.6	4.5	**NR**	4.4	**5.3**	**7.5**	4	2.7	3.1	4.3	4.5	**5.4**	3.43	**NR**	**6**	<5
	CMAP, mV	**3.0**	**2.3**	**1.4**	**NR**	**2.3**	4.4	**0.4**	17.1	**14.4**	11.5	8	4.2	4	10.3	**NR**	**2.9**	>3.5
	MCV, m/s	**29.5**	44.3	40.4	**NR**	40.4	**35.6**	**14.8**	43.8	45.4	50.7	41.7	**39.8**	49.6	43.8	**NR**	**32.1**	>40
Median sensory nerves	SNAP, μV	**2.8**	**2.6**	52.1	**11.6**	19.0	**NR**	**NR**	**NR**	57.6	35.7	20.4	**3.3**	17.2	17.2	37.4	**3.6**	>17
	SCV, m/s	**21.8**	60	64.7	**44.6**	62.1	**NR**	**NR**	**NR**	**10.1**	60.3	55.6	**47.3**	65	61.2	61.7	**41.5**	>50
Ulnar sensory nerves	SNAP, μV	**5.6**	**5.3**	44.3	**9.9**	**11.0**	**3.4**	**NR**	**NR**	**2.4**	33.6	19.6	**2.6**	17.8	19.2	31.4	**6.7**	>17
	SCV, m/s	51.6	56.5	65.3	**45.8**	53.4	**37.2**	**NR**	**NR**	55.6	55.2	61.7	**35.1**	50	54.5	67	**39.2**	>50
Sural nerves	SNAP, μV	**5.6**	**NR**	34.9	6.7	27.7	8.9	**NR**	26.2	7.4	15.4	**NR**	6.7	10	8.2	25.9	**NR**	>6
	SCV, m/s	51.6	**NR**	50.8	**33.5**	55.3	48.8	**NR**	**5.4**	43.4	57.2	**NR**	47.7	40.4	48.9	51.5	**NR**	>40

CMAP, compound muscle action potential; DML, distal motor latency; MCV, motor conduction velocity; NR, no response; SCV, sensory conduction velocity; SNAP, sensory nerve action potential.

The abnormal values were printed in bold.

Patient 4,15,19 did not undergo electromyography examination.

### Statistical analysis

The demographic, clinical and paraclinical data of patients were included for descriptive statistics. Variables were presented as mean standard deviation or median (quartile). Statistical analyses were performed using SPSS24 and Microsoft Excel. To assess potential association between factors and disease severity, we used Pearson or point-biserial correlation, and independent t-tests, depending on whether variables were continuous or categorical. P values of <0.05 were considered to be statistically significant. Given the exploratory nature of this study and the limited number of patients with anti-GD1a positivity, no formal sample size calculation was performed. The sample size (n=19) represents all consecutive eligible patients identified during the study period. Post-hoc, for a correlation coefficient of 0.5, our sample provides approximately 70% power at α=0.05 (two-tailed), indicating that moderate to strong associations can be detected, while weaker associations may be underpowered. Therefore, correlation analyses are presented as exploratory, and findings should be interpreted conservatively.

## Result

### Clinical manifestations

A total of 19 anti-GD1a antibody positive patients were included in this study, including 9 males and 10 females, with a mean age of 50.6 years (ranging from 18 to 75 years). The most common clinical features included absent tendon reflexes (84%), limb muscle weakness (84%), limb sensory impairment (58%), cranial nerve involvement (53%), cerebellar ataxia (32%), pain (26%) and autonomic nerve involvement (11%). Eleven patients (58%) had preceding infection (**Table 1**).

**Figure 1:**
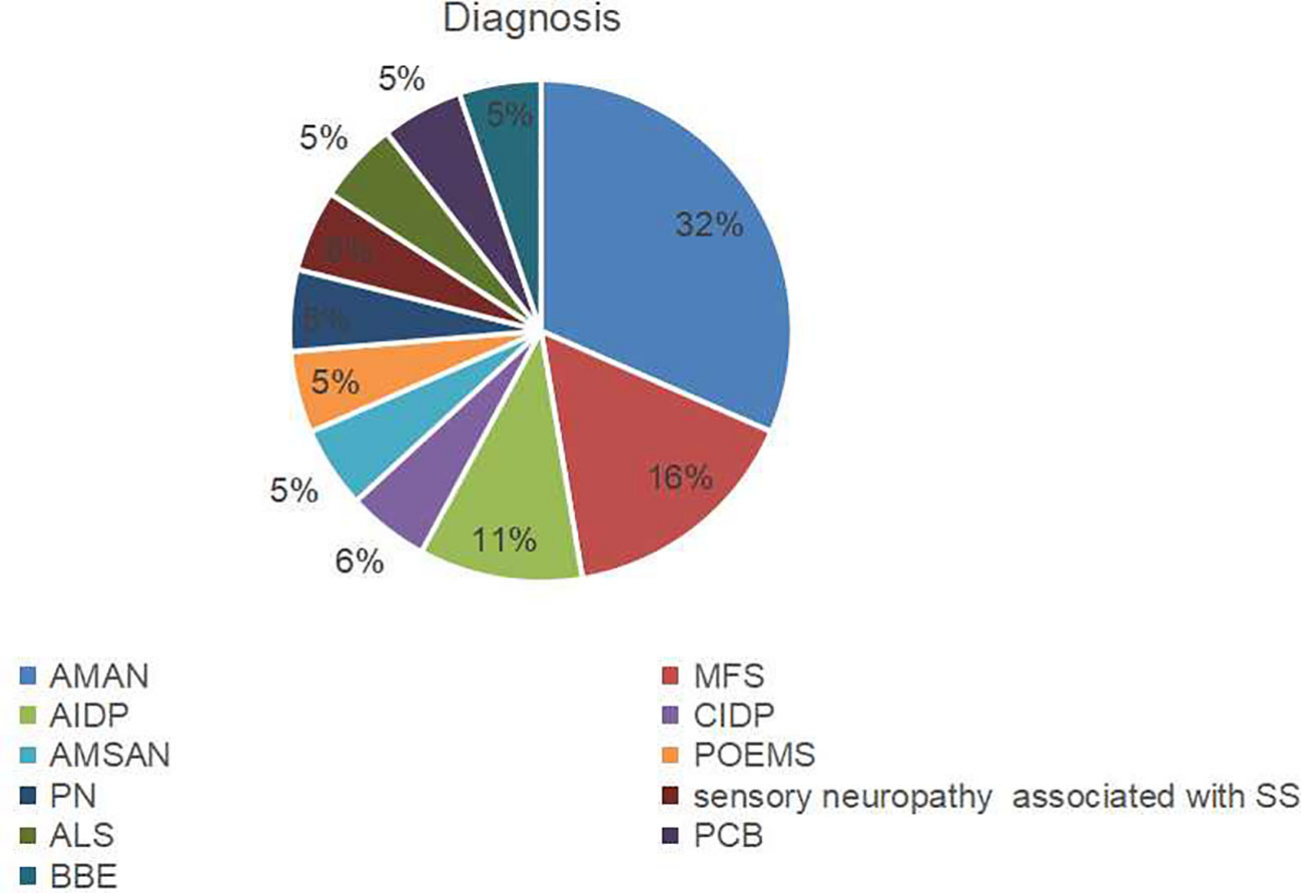
Diagnosis of patients seropositive for anti-GD1a antibody.

Fourteen patients seropositive for anti-GD1a antibody were acute course including AMAN(n=6), MFS(n=3), AIDP(n=2), and one case each of AMSAN, BBE and PCB variant. The mean MRC-sumscore and GBS disability score were 34.6(ranging from 6-60) and 3.4(ranging from 1-5), respectively. Five patients (36%) had mEGOS > 7, especially those with AMAN(5/6, 83%) or AMSAN(1/1, 100%), reflecting greater disease severity and poorer prognosis. The remaining five patients with anti-GD1a antibody were diagnosed as chronic disorders, including inflammatory demyelinating polyradiculoneuritis (CIDP)(n=1), amyotrophic lateral sclerosis (ALS) (n=1), POEMS syndrome(n=1), sensory neuropathy associated with Sjögren’s syndrome(n=1), and unexplained peripheral neuropathy (PN) (n=1) (**Figure 1**).

### Antibodies investigations

Of all 19 patients, five had anti-GD1a IgG or IgM antibodies exclusively. These patients were diagnosed with MFS, BBE, POEMS syndrome, sensory neuropathy associated with Sjögren’s syndrome, and unexplained PN, respectively. Fourteen patients had coexisting antibodies, as follows: anti-GQ1b IgG(n=6), anti-GT1a IgM or IgG(n=6), anti-GM1 IgM or IgG(n=5), anti-GD1b IgG (n=2), anti-GT1b IgG(n=2), anti-GM2 IgM (n=2), anti-GM3 IgM (n=3), anti-GM4 IgG (n=1), anti-GD3 IgG(n=1), and anti-Sulfatide IgM and IgG(n=1) (**Table 2**). Patients with anti-GQ1b or anti-GT1a antibodies were all classified as GBS. Five patients with anti-GM1 antibodies were diagnosed with AMAN(n=4) and CIDP(n=1).

### Other paraclinical investigations

Fourteen of 19 patients underwent lumbar puncture, half of them presented with albuminocytologic dissociation of CSF. The mean CSF cell counts was 2.5/mm^3^ (2 to 8). The mean CSF protein level was 0.71 ± 0.50 g/L(0.32 to 2.36 g/L). Sixteen of 19 patients underwent NCS, 9 (56%) exhibited typical axonal neuropathy characterized by significantly reduced CMAP amplitudes, 6 (38%) exhibited demyelinating neuropathy characterized by slow motor conduction velocities. One (6%) of them had normal nerve conduction findings. F wave conduction was performed in sixteen patients, and 10 (63%) showed abnormal results (16). (**Table 3**).

### Treatment and outcome of patients seropositive for anti-GD1a antibody

Two patients (11%) diagnosed with AMAN were admitted to the intensive care unit and required mechanical ventilation. Both patients were treated with intravenous immunoglobulins (IVIg) and weaned off ventilation finally. All 14 patients seropositive for anti-GD1a antibody with acute course(74%) were treated, including 7 (50%) with IVIg, 4 (29%)with methylprednisolone and IVIg, 2 (14%) with methylprednisolone and 1 (7%) with efgartigimod. Patients treated with IVIg received one dose of 2 g/kg administered over 5 days. The patient treated with efgartigimod was given intravenous dose of 10mg/kg once. Patients treated with methylprednisolone were given 0.5 g/day intravenously over 2 to 3 days and tapered off gradually. None of these 14 patients received maintenance therapy. Other five patients diagnosed with CIDP, POEMS syndrome, unexplained PN, Sjögren’s syndrome associated sensory neuropathy and ALS were treated with methylprednisolone, bortezomib, vitamin B12, methylprednisolone and riluzole, respectively.

After a mean 6 months follow-up, 8 of 14 (57%) patients seropositive for anti-GD1a antibody with acute course achieved complete neurological recovery and 6 (42.9%) had incomplete neurological recovery. No relapses were observed during follow-up.

### Association between key clinical variables, paraclinical parameters, and mEGOS

A moderate positive correlation was observed between cranial nerve involvement and higher mEGOSs (r=0.44), though it did not reach statistical significance (p=0.095). Albuminocytologic dissociation also showed a non-significant trend toward higher mEGOS scores (p = 0.087), suggesting it may be associated with worse prognosis in acute course. No statistically significant associations were found between other clinical features, serological profiles, or NCS subtypes and disease severity.

## Discussion

In this study, we characterized the clinical spectrum, antibody associations, electrophysiological and CSF features, and treatment responses in a Chinese cohort of 19 anti-GD1a antibody positive patients. We found patients with anti-GD1a antibody showed heterogeneous, most frequently were diagnosed as axonal variants of GBS, but also occurring in association with chronic neuropathies.

AMAN is the most common clinical phenotype in our cohort, similar to previous reports (4, 5). The high rate of preceding infections may further support the paradigm of molecular mimicry triggering an autoimmune response against neural antigens (17). The most common observed clinical manifestations are absent tendon reflexes, weakness and sensory impairment, which align with classical descriptions of immune-mediated neuropathies (18). The prevalence of cranial nerve damage and cerebellar ataxia suggests that anti-GD1a antibodies may be associated with a broader neurological phenotype beyond purely motor axonal involvement (6, 7).

Patients seropositive for anti-GD1a antibody with acute course tend to have higher GBS disability scores and mEGOSs, along with lower MRC-sumscores, especially in those with cranial nerve involvement or albuminocytologic dissociation, suggesting that GD1a antibody may serve as a potential marker of poor prognosis during the acute phase of disease. Further multicenter studies will be essential to confirm these associations.

Consistent with the findings of a previous study, another key finding is the frequent co-existence of other anti-ganglioside antibodies (2). Only 5 patients had isolated anti-GD1a antibodies, and they were associated with diverse neurological diseases. This finding cautions against making the diagnosis of GBS solely based on isolated anti-GD1a antibody positivity. In contrast, the presence of anti-GQ1b or anti-GT1a antibodies in 10 of 19 patients was invariably associated with the GBS diagnosis, consistent with the well-established clinical phenotypes of these antibodies (19–21). The clinical phenotype in patients with multiple antibodies specificities may be influenced more by coexisting antibodies. We note that in our cohort, patients with concomitant anti-GQ1b showed more frequent ophthalmoplegia (19, 22), while those with anti-GM1 had more pure motor axonal patterns (2, 23). This may suggest that the clinical presentation may be caused by the combined effects of multiple antibodies rather than by anti-GD1a antibodies alone. It should be noted that high titers tend to be pathogenic and low titers are more likely to be false positives or epiphenomena.

Paraclinical investigations provided diagnostic insights into the underlying pathophysiology. The finding of albuminocytologic dissociation in 50% of patients who underwent lumbar puncture is a hallmark of GBS and its variants, supporting the autoimmune mechanism (18). NCS revealed a spectrum of dysfunction: predominantly axonal and demyelinatin patterns. This electrophysiological heterogeneity mirrors the clinical diversity and indicates that patients seropositive for anti-GD1a antibody can present both axonal and demyelinating pathophysiology. The high rate (63%) of abnormal F wave conduction, indicating proximal motor nerve involvement, is consistent with typical GBS findings (3).

The treatment approach and outcomes differed markedly based on the course and diagnosis of patients seropositive for anti-GD1a antibody. For the acute course, immunotherapy with IVIg was the cornerstone of management, consistent with standard international guidelines (3). The successful weaning of two severely affected AMAN patients from mechanical ventilation highlights the efficacy of early immunomodulation (24). The favorable prognosis in the acute group—with 57% achieving complete neurological recovery within a mean of 6 months and no observed relapses—is encouraging and may suggests these cases typically follow a monophasic course (18). In contrast, the five patients with chronic neuropathies (CIDP, ALS, etc.) received disease-specific therapies and some of them achieve partial remission, highlighting that the presence of anti-GD1a antibodies in such settings may represent an epiphenomenon or suggest a modulatory role in disease pathogenesis, warranting further investigation (1).

Although our study provide new insights into the understanding of patients seropositive for anti-GD1a antibody, several limitations should be acknowledged. First, the relatively small sample size and the retrospective descriptive nature of the analysis limit the strength of the conclusions. Second, the relatively short follow-up period for the chronic cases precludes long-term prognostic conclusions. Third, there are selection bias such as single-center retrospective design and only patients who underwent antibody testing were included. Lack of standardized physiotherapy protocols and differences in coexisting antibody profiles are confounders. Lastly, our findings were adult-only and may not apply to pediatric populations.

In summary, patients seropositive for anti-GD1a antibody have a wide clinical spectrum, from acute GBS variants to chronic neuropathies. The clinical phenotype and disease severity is likely influenced by the anti-GD1a antibody itself and modulated by coexistence of other ganglioside antibodies. Patients seropositive for anti-GD1a antibody with acute course respond favorably to immunotherapy with an overall good prognosis. Future prospective studies with larger cohorts are needed to define the precise pathogenic role of anti-GD1a antibodies.

## Data Availability

The raw data supporting the conclusions of this article will be made available by the authors, without undue reservation.
